# Ethylene promotes fruit ripening initiation by downregulating photosynthesis, enhancing abscisic acid and suppressing jasmonic acid in blueberry (*Vaccinium ashei*)

**DOI:** 10.1186/s12870-024-05106-4

**Published:** 2024-05-18

**Authors:** Yi-Wen Wang, Savithri U. Nambeesan

**Affiliations:** 1 Department of Horticulture, University of Georgia, 1111 Miller Plant Sciences Building, Athens, GA 30602 USA; 2 Center for Applied Genetic Technologies, University of Georgia, 111 Riverbend Road, Athens, GA 30602 USA; 3 Institute of Plant Breeding, Genetics & Genomics, University of Georgia, 111 Riverbend Road, Athens, GA 30602 USA

**Keywords:** Fruit development, Auxin, RNA-seq, Transcriptomics, Hormone, Ethephon

## Abstract

**Background:**

Blueberry fruit exhibit atypical climacteric ripening with a non-auto-catalytic increase in ethylene coincident with initiation of ripening. Further, application of ethephon, an ethylene-releasing plant growth regulator, accelerates ripening by increasing the proportion of ripe (blue) fruit as compared to the control treatment. To investigate the mechanistic role of ethylene in regulating blueberry ripening, we performed transcriptome analysis on fruit treated with ethephon, an ethylene-releasing plant growth regulator.

**Results:**

RNA-Sequencing was performed on two sets of rabbiteye blueberry (‘Powderblue’) fruit: (1) fruit from divergent developmental stages; and (2) fruit treated with ethephon, an ethylene-releasing compound. Differentially expressed genes (DEGs) from divergent developmental stages clustered into nine groups, among which cluster 1 displayed reduction in expression during ripening initiation and was enriched with photosynthesis related genes, while cluster 7 displayed increased expression during ripening and was enriched with aromatic-amino acid family catabolism genes, suggesting stimulation of anthocyanin biosynthesis. More DEGs were apparent at 1 day after ethephon treatment suggesting its early influence during ripening initiation. Overall, a higher number of genes were downregulated in response to ethylene. Many of these overlapped with cluster 1 genes, indicating that ethylene-mediated downregulation of photosynthesis is an important developmental event during the ripening transition. Analyses of DEGs in response to ethylene also indicated interplay among phytohormones. Ethylene positively regulated abscisic acid (ABA), negatively regulated jasmonates (JAs), and influenced auxin (IAA) metabolism and signaling genes. Phytohormone quantification supported these effects of ethylene, indicating coordination of blueberry fruit ripening by ethylene.

**Conclusion:**

This study provides insights into the role of ethylene in blueberry fruit ripening. Ethylene initiates blueberry ripening by downregulating photosynthesis-related genes. Also, ethylene regulates phytohormone-metabolism and signaling related genes, increases ABA, and decreases JA concentrations. Together, these results indicate that interplay among multiple phytohormones regulates the progression of ripening, and that ethylene is an important coordinator of such interactions during blueberry fruit ripening.

**Supplementary Information:**

The online version contains supplementary material available at 10.1186/s12870-024-05106-4.

## Background

Ripening is an important developmental phase that renders the fruit attractive, and imparts flavor making the fruit palatable. Important physiological events that occur during ripening include an increase in sugar to acid ratio, changes in cell wall metabolism, and synthesis of specific pigments and flavor volatiles [1]. Although all fleshy fruits display the above changes, fruits are often classified according to their ripening physiology into two types: climacteric and non-climacteric fruits. Climacteric fruits display an increase in respiration during ripening, whereas such an increase is not discernable in non-climacteric fruits. Further, ethylene production is autocatalytic and an increase in ethylene production during ripening is critical in facilitating ripening-related changes in multiple climacteric fruits such as tomato (*Solanum lycopersicum*) [2], banana (*Musa sp.*) [3], peach (*Prunus persica*) [4, 5], and apple (*Malus × domestica*) [6]. Ethylene induces the expression of cell wall loosening enzymes such as polygalacturonase in tomato [7], apple [8], and kiwifruit (*Actinidia chinensis*) [9]. In apple, a protein involved in ethylene signaling, ETHYLENE INSENSITIVE 3 (EIN3)-LIKE 1 (MdEIL1), increases the transcript abundance of *MdMYB1*, a transcription factor that promotes anthocyanin biosynthesis [10]. In pear (*Pyrus communis* L.), ETHYLENE RESPONSE FACTORS (ERFs) regulate the MYB and bHLH (basic helix-loop-helix) transcription factors, to promote anthocyanin biosynthesis [11]. Even though the role of ethylene is not clear in non-climacteric fruits, it may yet be involved in promoting specific aspects of ripening. For example, in strawberry (*Fragaria × ananassa*), ethylene regulates activities of the cell wall degrading enzymes, polygalacturonase and β-galactosidase, although it does not alter *EXPANSIN2* gene expression [12, 13]. Further, ethylene promotes fruit color development in strawberry [13]. In grape (*Vitis vinifera*), treatment with 1-methylcyclopropene (1-MCP), which inhibits ethylene perception also inhibits the expression of sugar transport related genes [14].

Abscisic acid (ABA) may play a larger role, in comparison to that of ethylene, in facilitating ripening in non-climacteric fruit [15]. A peak in ABA concentration and signaling-related gene expression are noted at the onset of ripening in grape [16, 17]. External application of ABA after *véraison* in grape promotes color development, but does not affect fruit firmness, pH, total soluble solids (TSS), and titratable acidity (TA) [18]. Application of ABA followed by transcriptome analysis in grape suggests downregulation of photosynthesis, autocatalysis of ABA synthesis, and stimulation of pigment biosynthesis [19]. Application of ABA promotes ripening in strawberry and induces expression of cell wall degradation-related genes [20]. In climacteric fruits such as tomato, ABA concentration increases during ripening and precedes the climacteric rise in ethylene evolution [21]. Further, application of ABA accelerates ripening and increases the transcript abundance of certain members of the ethylene biosynthesis gene families including 1-aminocyclopropane-1-carboxylic acid (ACC) synthase (*ACS*) and ACC oxidase (*ACO*), suggesting that ABA plays a positive role upstream of ethylene during fruit ripening [21, 22]. Conversely, when ethylene signaling is blocked using 1-MCP, ABA accumulation is delayed compared to that in the control and ABA treatments alone, suggesting that ethylene regulates ABA accumulation [22]. Thus, interplay between these two hormones may, at least in climacteric fruits, influence progression of ripening.

In addition, auxin can negatively regulate ripening as suggested by pharmacological and transgenic approaches. Generally, auxin regulates processes during early fruit development such as promotion of fruit growth by influencing cell division and elongation, synergistically with cytokinin and gibberellin [15]. In grape, auxin levels are high during early fruit development and its concentration decreases dramatically during ripening [23]. In strawberry, auxin is synthesized in the achenes and promotes the expansion of the receptacle during fruit development, and its levels diminish during ripening [24]. Thus, auxin is considered as a negative regulator of fruit ripening. Auxin application delays ripening in strawberry, grape and tomato [25–28]. Treatment with auxin downregulates genes involved in cell wall modification, sugar metabolism and anthocyanin pigmentation in grape and strawberry [20, 27, 29]. Similarly, in tomato, auxin treatment downregulates genes associated with cell wall degradation and carotenoid biosynthesis [30]. Treatment of grape with auxin at the pre-*véraison* stage indicates that although transcript abundance of ethylene biosynthesis-related genes is enhanced, general downregulation of ethylene induced ripening processes such as cell wall metabolism and pigment biosynthesis occur, suggesting an antagonistic interaction of auxin and ethylene in this process [31]. After cherry fruit tomato are treated with 0.45 mM 2,4-D (an auxin) at the Mature Green stage, the climacteric rise in ethylene is decreased and delayed by almost 3 d, and is associated with repression of ethylene biosynthesis-related gene expression [30]. When ethylene perception is blocked by 1-MCP in apple, it de-represses auxin-regulated genes which in-turn activate ethylene biosynthesis to restore ethylene homeostasis [32, 33] suggesting that auxin may yet play a promoting role in initiating ethylene biosynthesis during ripening. Further, expression of auxin-inducible transcription factors, auxin response factors (ARFs), in apple (*MdARF5*) and peach (*PpARF6*), promoted ethylene biosynthesis [34–36]. In tomato, silencing of the paralogs, *SlARF2A* and *2B* led to a decrease in climacteric ethylene production and impairment in ripening [37]. Further, overexpression of *ARF2A* in tomato, resulted in accelerated ripening. *ARF2A* expression is inducible by auxin, ethylene and ABA suggesting that this protein potentially interconnects multiple hormonal pathways to initiate fruit ripening [38]. Therefore, these studies suggest that auxin may be involved in positively regulating initiation of ripening. Increased ethylene production led to a decline in auxin levels, likely due to ethylene induced activation of auxin conjugating enzyme, that increased inactive IAA-Asp conjugate accumulation [35], further suggesting crosstalk between these two hormones and a feedback loop.

Ripening physiology in blueberry is not well characterized. Previously, our work indicated that blueberry fruit exhibit atypical climacteric ripening physiology as they display a respiratory climacteric and ethylene production comparable to that in climacteric fruit [39]. However, the rise in ethylene evolution during ripening is developmentally controlled and not autocatalytic. Further, ethylene signaling components are functional during blueberry fruit ripening [39]. An evaluation of 12 highbush blueberry cultivars indicated variation in ethylene production during fruit ripening across genotypes [40]. In this study, we further explored the role of ethylene in blueberry ripening by performing transcriptome analysis on fruit treated with ethephon (2-chloroethane phosphonic acid), an ethylene releasing plant growth regulator (PGR). Transcriptomes of fruit harvested at 1 day and 2 days after ethephon treatment were compared to that of the developing fruit transcriptomes to determine the processes altered by ethylene during blueberry ripening.

## Results

### Effect of ethephon application on fruit ripening in blueberry

**Fig. 1:**
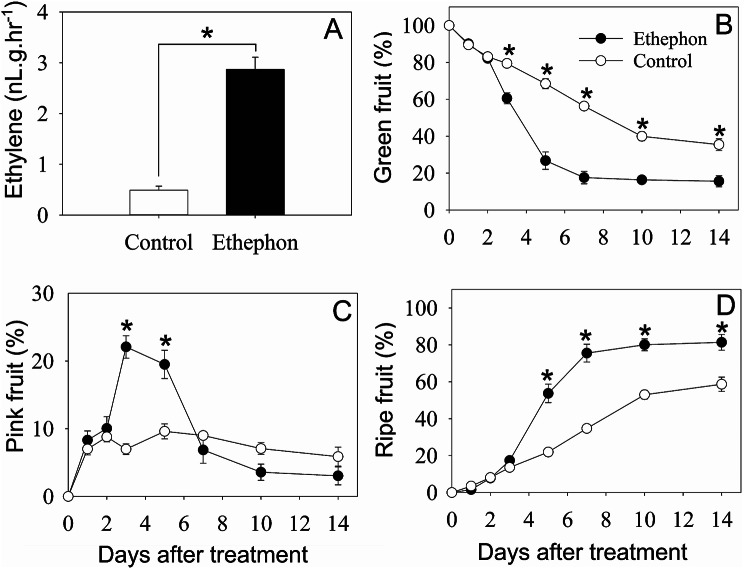
Response of rabbiteye blueberry ‘Powderblue’ to preharvest treatments with water (control) and ethephon. Ethylene production of blueberry fruit 2 days after treatment (**A**). Percentage of ripening based on skin color of blueberry fruit after treatments (**B**, **C**, **D**). Asterisks indicate significant differences between treatments within a given day after treatment

Ethephon application resulted in a 6-fold increase in ethylene evolution compared to the control at 2 days after treatment (Fig. 1A). The effect of ethephon on ripening could be observed as early as 3 days after treatment. Ethephon treated fruit displayed a lower proportion of Green fruit than that in the control from 3 days after treatment (Fig. 1B). The proportion of Pink fruit increased by 3- and 2-fold in the ethephon treatment in comparison to the control at 3 and 5 days after treatment, respectively, while the proportion of ripe fruit was significantly higher (by 1.25- to 2.5-fold) after ethephon treatment from 5 days after treatment (Fig. 1C, D).

### Transcriptome sequencing

In total, 796 million raw reads were generated, with an average of 38 million reads in each sample. After trimming out adapters and low quality-reads, 771 million reads remained, with an average of 36.8 million reads in each sample. Subsequently, 81.8% of trimmed reads were mapped to the fruit-specific transcriptome [41], with 55.9% uniquely mapped reads and 25.8% multi-mapping reads (Table S1).

**Fig. 2:**
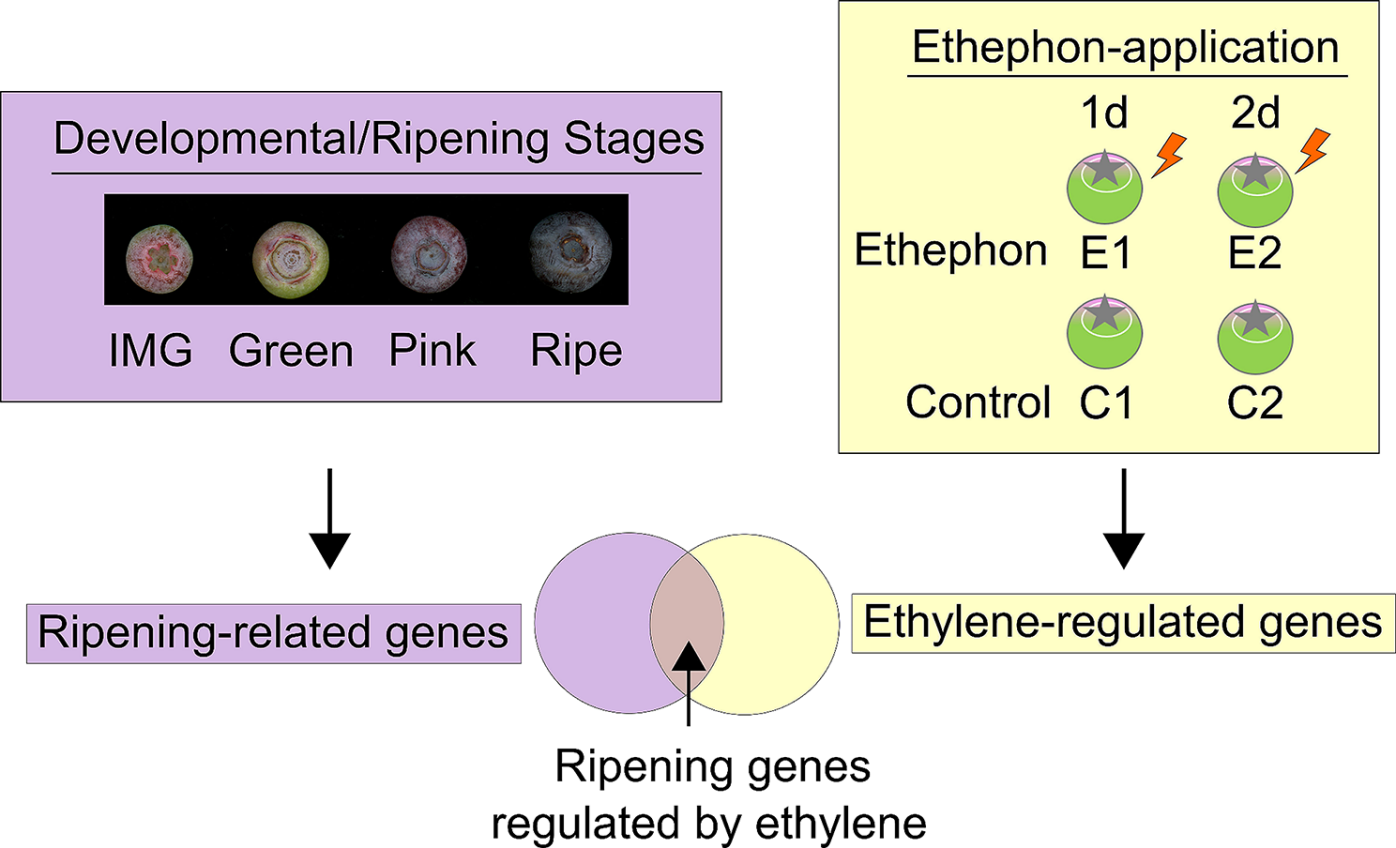
Experimental design for transcriptome analysis. For transcriptome analyses, four developmental stages that included, Immature green (IMG), Green, Pink, and Ripe fruit were used to identify ripening-related genes. Ethylene-regulated genes were identified by comparing transcriptomes of ethephon (E) and control (C) treated fruit at 1 and 2 days after treatment. Ripening-related genes regulated by ethylene were identified, by comparing the overlap of ripening-related and ethylene-regulated genes

Transcriptome analyses were performed from four developmental/ripening stages (Immature Green [IMG], Green, Pink, and Ripe fruit) to identify ripening-related genes (Fig. 2). Ethylene-regulated genes were identified by comparing transcriptomes of ethephon and control treated fruit (Fig. 2). Finally, by comparing the overlap of ripening-related and ethylene-regulated genes, ripening-related genes regulated by ethylene were identified (Fig. 2). Since underlying gene expression differences precede phenotypic differences (evident at 3 days of treatment as changes in the proportion of Green and Pink fruit), ethephon and control treated fruit at 1 and 2 days after treatment were used in this study.

To obtain an overview of the transcriptomes among all samples, transcript profiles using log_2_-fold-changes between each pair of samples were visualized by multidimensional scaling (MDS) plot analysis (Fig. S1). The higher the distance between each pair of samples the greater the dissimilarity of their transcriptomes. Overall, all replicates within a given ripening stage, and control and ethephon treated fruit at each day after treatment clustered together (Fig. S1) indicating robustness of sample collection and quality. Furthermore, the separation of each ripening stage was distinct, with the IMG, Green, Pink and Ripe fruit stages being separated along the x-axis. Overall, the variation within control fruit at day 1 and 2 were smaller with similar variability between days after ethephon treatments (Fig. S1). Ethephon-treated fruit showed a shift in transcriptome profile towards the right along the X-axis and closer to the Green stage compared to the control fruit (Fig. S1), suggesting progression towards ripening initiation following ethephon treatment.

### Transcriptome analysis reveals global gene expression patterns during blueberry ripening

The differentially expressed genes (DEGs) between each pair of ripening stages were identified with the cutoff of < 0.01 false discovery rate and ≥ 2-fold change. There were 2,019, 1,009, and 1,663 DEGs in the IMG vs. Green, Green vs. Pink, and Pink vs. Ripe comparisons, respectively (preceding stage as control) (Table 1). The numbers of upregulated and downregulated genes were similar between IMG and Green fruit (1,108 and 911, respectively) (Table 1). There were more downregulated genes (601) than upregulated genes (408) between Green and Pink fruit. However, there were about 2-fold more upregulated genes (1,156) than downregulated genes (508) between Pink and Ripe fruit (Table 1).

**Table 1:** Number of differentially expressed genes (DEGs) during ripening and after ethephon treatment in rabbiteye blueberry ‘Powderblue’. IMG: immature green; ED1, ED2: ethephon day1 and day 2; CD1, CD2: control day 1 and day 2

Stage/treatment	Comparison	Total	Up	Down
Ripening stage	Green vs. IMG	2019	1108	911
	Pink vs. Green	1009	408	601
	Ripe vs. Pink	1663	1156	508
Ethephon treatment	ED1 vs. CD1	625	107	518
	ED2 vs. CD2	260	70	190

**Fig. 3:**
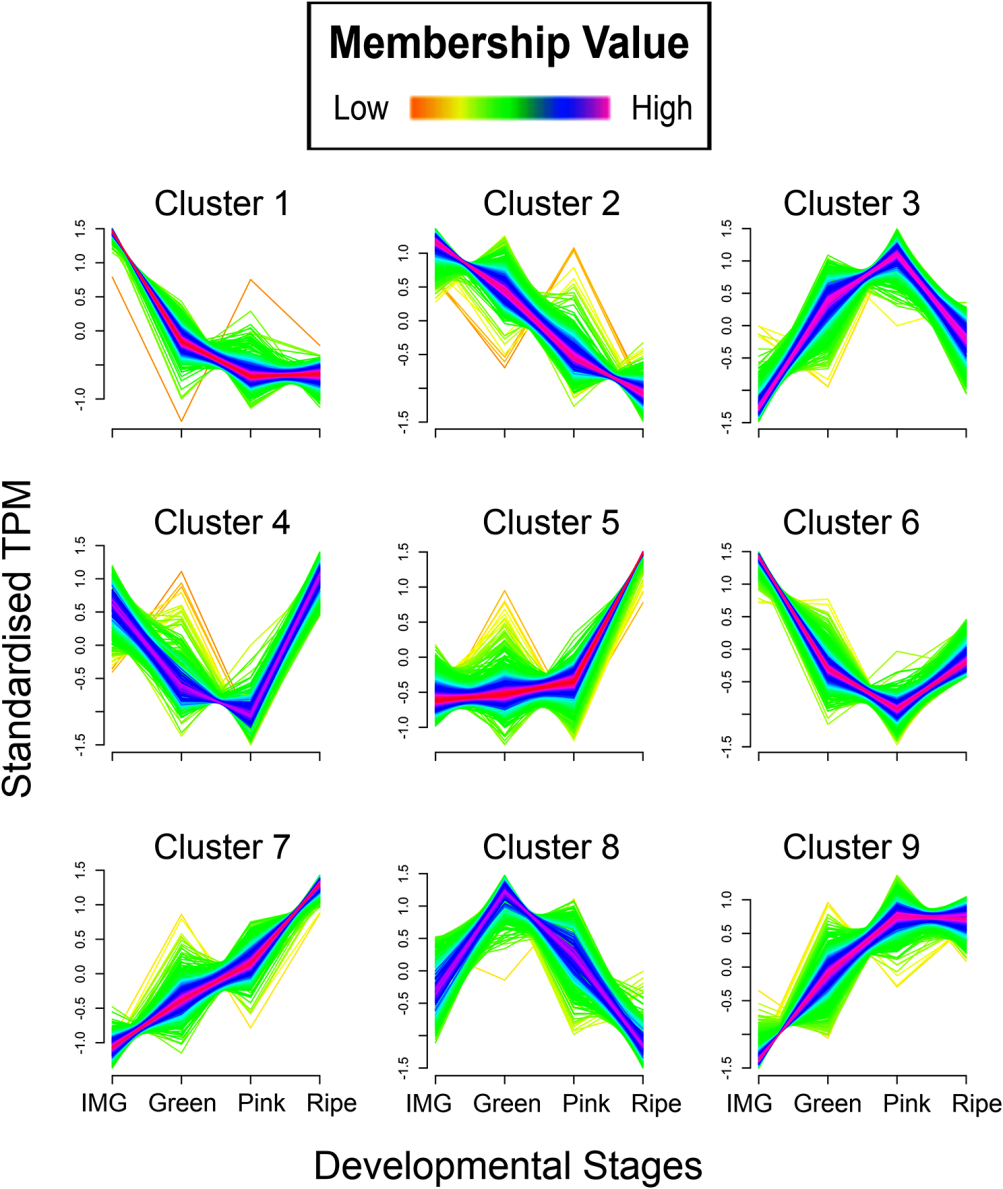
Cluster analysis of differentially expressed genes during ripening using Mfuzz. Each line indicates the scaled expression pattern of a gene, with colors from purple to orange showing the fitness of the gene in the cluster from high to low

**Fig. 4:**
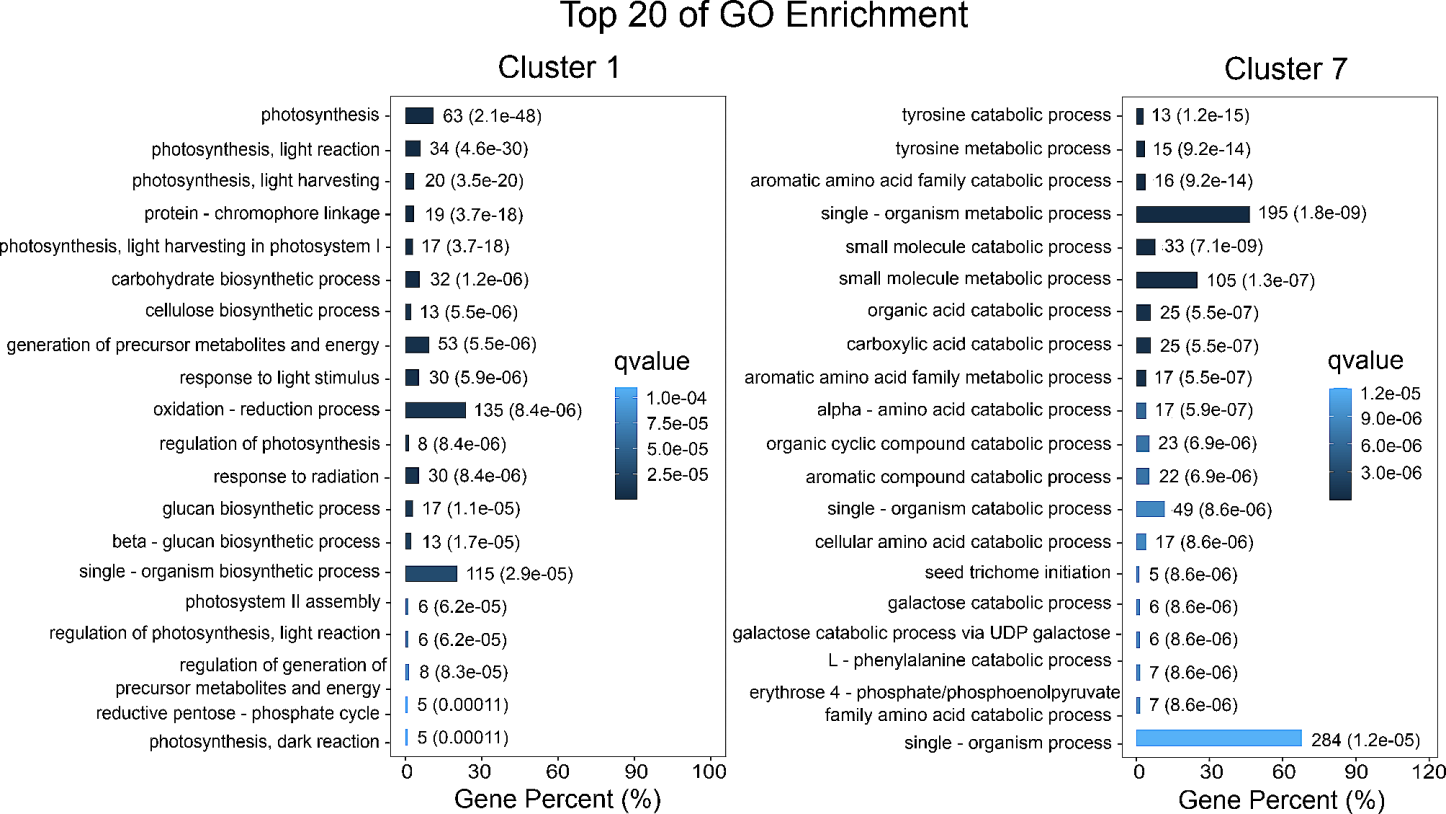
Top 20 enriched biological process gene ontology (GO) terms for ripening-related genes in cluster 1 and cluster 7

Temporal expression patterns during ripening were analyzed by categorizing all the DEGs into clusters with similar expression patterns. Clustering analysis resulted in nine clusters with distinct expression patterns (Fig. 3). The membership of genes in each cluster from 1 to 9 were 1,095, 564, 451, 326, 883, 677, 698, 329, and 603, respectively (Table 2; Table S2). Gene ontology (GO) enrichment analysis for each cluster was performed and the top 20 GO enrichment terms in biological process category, based on Q value in each cluster were determined (Fig. S2). In cluster 1, photosynthesis related terms were most evident, and these transcripts decreased in abundance dramatically between IMG to the Pink stage and remained similar until the Ripe stage (Fig. 4; Fig. S2). In cluster 2, transcripts associated with amide and peptide biosynthesis processes, and translation were enriched and downregulated gradually during ripening (Fig. S2). Genes contained in cluster 3 gradually increased from IMG to Pink stage and then decreased from Pink to Ripe stage. The GO enrichment terms associated with cluster 3 genes were associated with plant cell wall organization and carbohydrate metabolic processes (Fig. S2). In cluster 4, abiotic stress-related terms were enriched, and associated transcripts displayed a general downregulation during ripening initiation until the Pink stage followed by upregulation between the Pink and Ripe stages (Fig. S2). In cluster 5, transcripts associated with protein folding, aminoglycan and chitin processes were enriched and upregulated during ripening especially during the Pink and Ripe stages (Fig. S2). In cluster 6, overall patterns indicated downregulation from the IMG to Pink stage and slight upregulation between the Pink and Ripe stages. Transcripts associated with this cluster included chloroplast RNA processing, homogalacturonan biosynthetic process and hexose transmembrane transport, with only a few genes under each of these terms (Fig. S2). The transcripts in cluster 7, increased during fruit ripening and were associated with tyrosine, and aromatic amino acid family catabolic process (Fig. 4; Fig. S2). In cluster 8, transcripts displayed upregulation between IMG and Green stages followed by downregulation between the Green and Ripe stages. The GO enriched categories in this cluster included those associated with protein folding, and endoplasmic reticulum (ER) unfolded protein response (Fig. S2). In cluster 9, flavonoid biosynthesis and aromatic amino acid family metabolic process were enriched (Fig. S2), with transcripts displaying an increase between IMG to Pink stage and then remaining constant between the Pink and Ripe stages.

**Table 2:** Summary of the numbers of total, and ethephon-regulated genes within each cluster

Cluster	Total	Upregulated				Downregulated		
		Day 1	Day 2	Total		Day 1	Day 2	Total
Cluster1	1095	2	1	3		351	143	356
Cluster2	564	1	0	1		18	6	22
Cluster3	451	27	18	38		0	0	0
Cluster4	326	1	0	1		26	7	33
Cluster5	883	13	8	20		14	5	17
Cluster6	677	0	0	0		90	25	95
Cluster7	698	12	8	17		0	0	0
Cluster8	329	25	22	34		2	0	2
Cluster9	603	19	10	24		1	0	1

### Ethylene-regulated ripening-related genes

There were 625 and 260 DEGs in response to ethephon at day 1 and day 2 after treatment (Table 1), respectively, indicating differential regulation by ethephon occurred by day 1 after treatment. Of these DEGs, there were 107 and 70 upregulated genes and 518 and 190 downregulated genes on day 1 and day 2, respectively after ethephon treatment (Table 1), indicating that ethephon regulates ripening primarily through downregulation of gene expression.

**Fig. 5:**
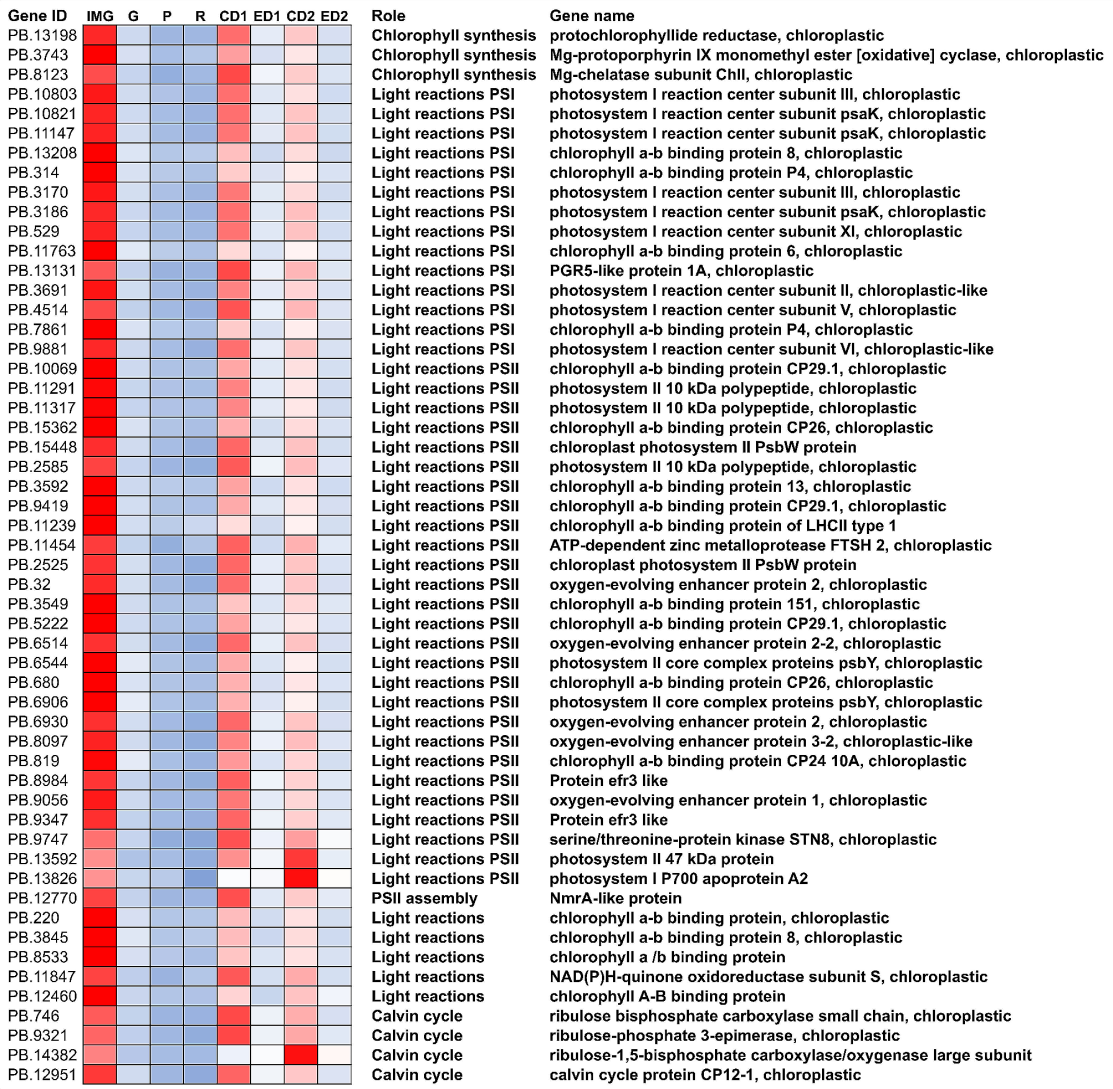
Gene expression levels of photosynthesis-related genes in Cluster 1 that overlaps with ethephon treatment. IMG: immature green, CD1, CD2: control treated fruit after 1 and 2 days of treatment, ED1, ED2: ethephon treated fruit after 1 and 2 days of treatment. The gene expression level was normalized within each gene. Red to blue indicates a gradient of high to low level of expression based on transcript per million (TPM) values

The ethylene-regulated ripening genes were identified by determining the overlap between transcripts upregulated/downregulated after day 1 and 2 of ethephon treatment with each of the nine ripening clusters. The greatest number of genes downregulated upon ethephon treatment overlapped with Cluster 1 (356 transcripts; 32% of all transcripts in Cluster 1) and Cluster 6 (95 transcripts; 7% of all transcripts in Cluster 6) (Table 2). Genes in these two clusters were mostly downregulated between IMG and Green stage. In Cluster 1, ripening-related genes downregulated at day 1 and 2 after ethephon treatment were mainly photosynthesis-related genes (Table S3; Fig. 5). When a search for ‘photosynthesis’ was performed for GO terms in Cluster 1, a total number of 64 transcripts were identified, of which 54 transcripts were differentially regulated by ethephon (Fig. 5). These transcripts had functions associated with the Calvin cycle, and light reactions in photosystem I and photosystem II (Fig. 5). In Cluster 6, after ethephon treatment, transcripts related to several processes were downregulated including several genes related to carbohydrate metabolism (Table S3).

The top 2 clusters overlapping with ethephon upregulated genes were cluster 3 and cluster 8, which had 38 (11% of all transcripts in Cluster 3) and 34 (10% of all transcripts in Cluster 8) overlapping genes respectively (Table 2). Genes in these two clusters were upregulated between IMG to Green (Fig. 3). Ripening-related genes upregulated by ethephon in these two clusters included hormone-related genes such as auxin conjugation gene (*INDOLE-3-ACETIC ACID-AMIDO SYNTHETASE*), auxin responsive gene (*auxin-responsive protein SAUR76-like*), abscisic acid (ABA) signaling gene (*abscisic acid receptor PYR1-like*), and ethylene signaling genes (*REVERSION-TO-ETHYLENE SENSITIVITY1*; *ARGOS-like protein*). In addition, in these two clusters, several cell wall modification genes (*probable pectate lyase 8*; *expansin-A4*), and sugar metabolism related genes (*galactinol synthase 2-like*; *sugar transporter ERD6-like 16*) were upregulated by ethephon (Table S3).

### Hormone analysis and related DEGs during fruit ripening

As ethylene-induced, hormone-related genes were identified in our analysis of upregulated genes upon ethephon treatment, we further determined the subset of ethylene-induced hormone-related genes by using a key word search. Overall, the number of DEGs associated with ABA, auxin, ethylene and JA were 99, 63, 52, and 30 respectively during ripening (Table S5). Of the total number of genes, ethephon affected the abundance of approximately 13%, 10%, 17%, and 17% of transcripts related to ethylene, ABA, auxin, and JA metabolism and signaling, respectively (Table S5).

**Fig. 6:**
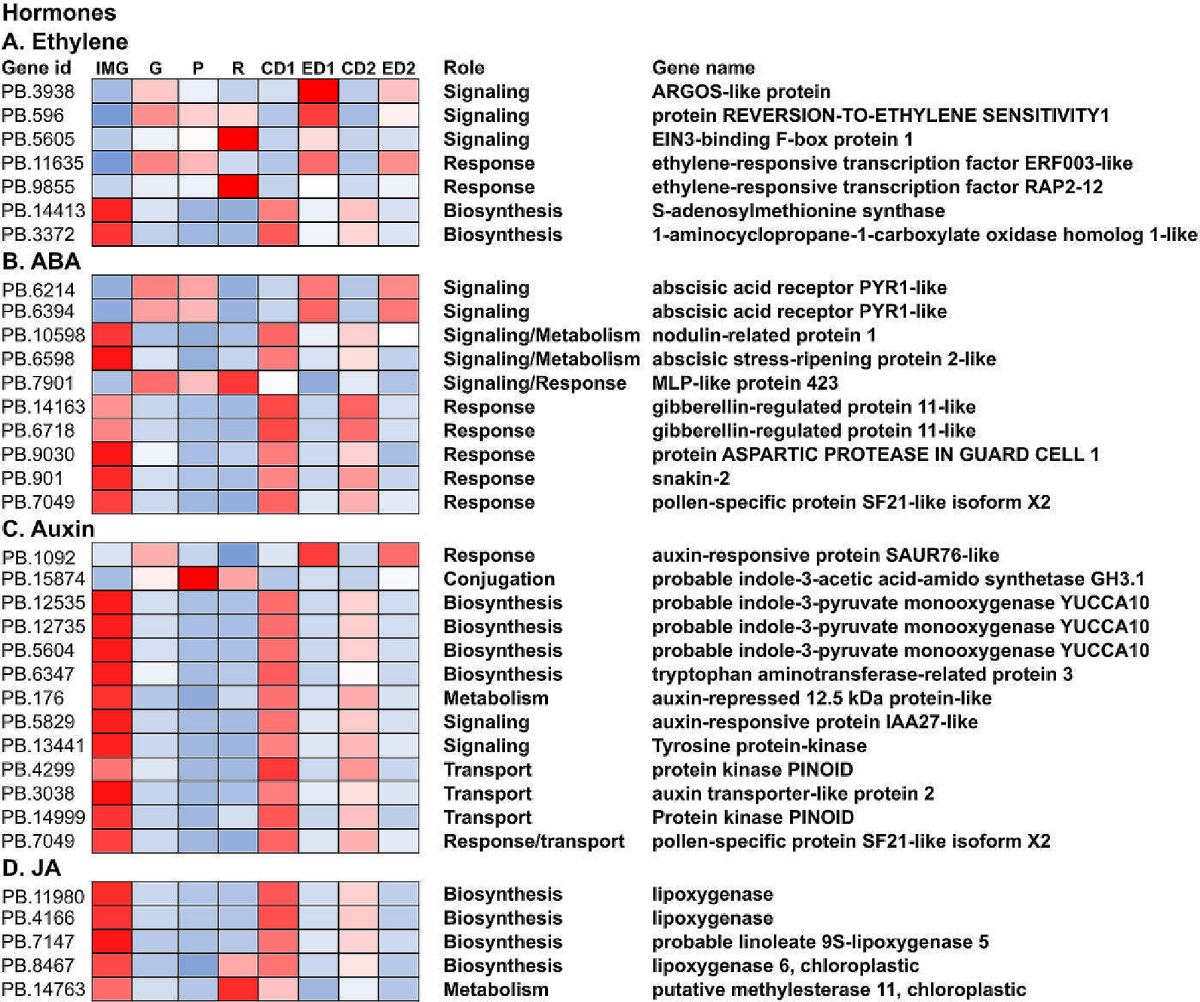
Gene expression levels of hormone related genes in the ethylene (**A**), ABA (**B**), auxin (**C**) and JA (**D**) metabolism. Red to blue indicates a gradient of high to low levels of expression based on transcript per million (TPM) values

Of the seven ethylene-related genes that were differentially regulated after ethephon treatment, two biosynthesis-related genes were downregulated, and five signaling and response genes were upregulated (Fig. 6A). In relation to ABA, two receptor genes were upregulated and 8 response and signaling genes were downregulated after ethephon treatment (Fig. 6B). In relation to auxin, of the 11 downregulated genes, four each were related to biosynthesis and transport, and of the three upregulated genes, one was involved in conjugation (Fig. 6C). In relation to JA acid-related genes, all five genes were downregulated by ethephon, four of which were involved in biosynthesis (Fig. 6D).

**Fig. 7:**
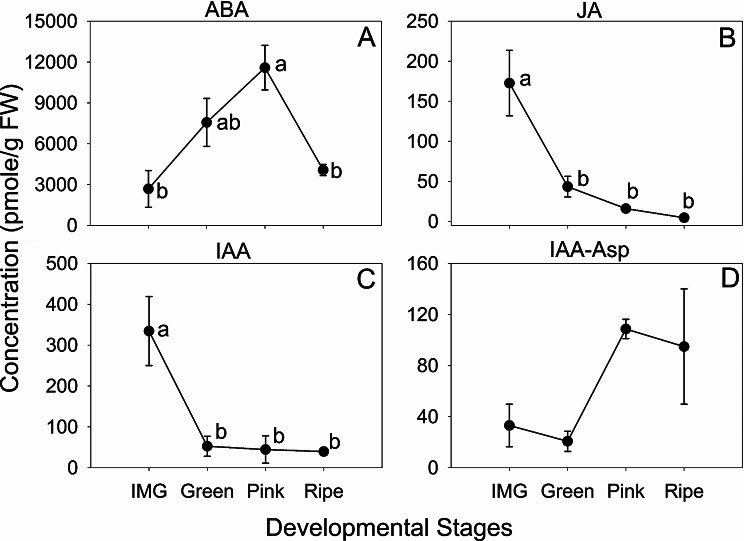
Phytohormone concentrations during blueberry fruit development. ABA (**A**), JA (**B**), IAA (**C**) and IAA-Asp (**D**) concentrations during fruit ripening at Immature green (IMG), Green, Pink and Ripe stages are presented. Same letter above symbols indicates that the corresponding stages are not significantly different from each other based on one-way analysis of variance (α = 0.05) and Tukey’s HSD

**Fig. 8:**
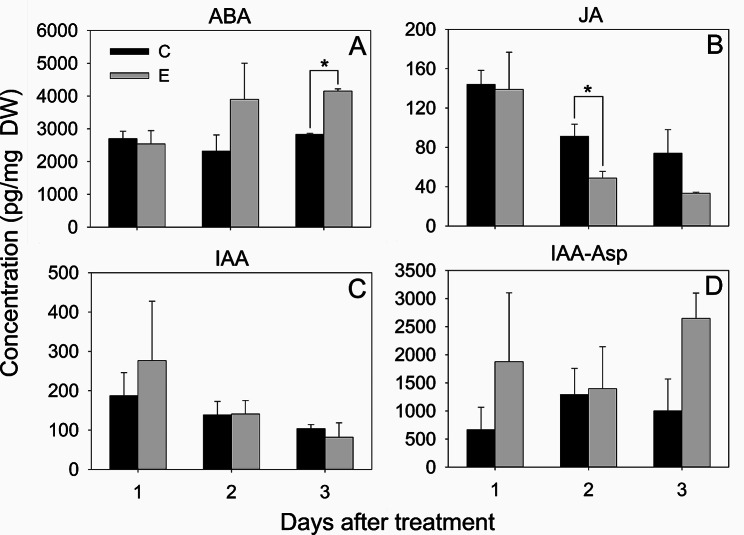
Effect of ethephon applications on phytohormone concentration in blueberry fruit. Asterisk indicates significant difference (α = 0.05) between the control and ethephon treated samples within a given day after treatment based on Student’s t-test

We further analyzed the roles of phytohormones by quantifying their concentrations. ABA concentrations gradually increased by 4.3-fold from IMG to Pink stage and decreased thereafter as ripening progressed (Fig. 7A). JA concentrations decreased by 4-fold from IMG to Green stage (Fig. 7B). Similarly, IAA also decreased by 6.4-fold during IMG and Green stage transition, while IAA-Asp levels showed an increasing trend by 3.3-fold between the Green and Pink stage (Fig. 7C, D). Ethephon treatment increased ABA concentration by 1.5-fold at day 3 and reduced JA concentration by around 2-fold at day 2 compared to the control treatment (Fig. 8A, B). Ethephon treatment did not affect IAA and IAA-Asp concentrations, however IAA-Asp displayed an increased trend at day 3 after ethephon treatment (Fig. 8C, D). In addition, ABA-GE, MeJA, Ile-JA-1, and Ile-JA-2 were also quantified after ethephon treatment. While Ile-JA-1 was not detectable, Ile-JA-2 was only detectable at day 0 (before treatment), MeJA and ABA-GE were not significantly different between control and ethephon treated fruit (data not shown).

## Discussion

Genes differentially regulated between the IMG and Ripe stages could be grouped into nine clusters, each showing distinct patterns of expression during blueberry fruit ripening. Cluster 1, with the highest number of DEGs, included GO enriched categories related to photosynthesis. These results indicate downregulation of photosynthesis during the onset of blueberry ripening. Such a downregulation in photosynthesis-related genes has also been noted in multiple fruit crops such as strawberry, grape and peach [42–44]. During ripening, tomato fruit transitions from being a partly autotrophic organ to a heterotrophic organ exhibiting downregulation of photosynthesis-related genes. In tomato, fruit photosynthesis during the early phase of fruit growth, although not essential, can contribute to about 10–20% of the total fruit carbon (C) [45, 46] with the remaining requirement supported by imported C. Thus, downregulation of photosynthesis during ripening initiation appears to be consistent across multiple fruits. In blueberry, fruit photosynthesis can contribute up to 85% of its C requirements during very early stages development from 5 to 10 days after anthesis. This value decreases substantially during later stages, with up to 15% C being supported *via* fruit photosynthesis throughout fruit development [47]. Consistently, components of the photosystem reaction centers, and those associated with electron transport and the Calvin cycle are downregulated at the onset of ripening [48]. In the current study, comprehensive analyses of fruit development clearly support these previous studies and indicate that downregulation of photosynthesis is concomitant with the onset of ripening in blueberry. Coincidently, genes involved in chlorophyll degradation (TPM value > 50) such as *PHEOPHORBIDE A OXYGENASE* (Cluster 9) and *PROTEIN STAY-GREEN* (Cluster 3) displayed higher expression during ripening (Table S6). Consistently, breakdown of chlorophyll *via* pheophorbide a oxygenase is an important event during fruit ripening in apple [49, 50].

Ethephon-treated fruit displayed progression to the Green stage fruit suggesting that ethylene promotes rapid transcriptomic changes reflective of ripening initiation. These results were also supported by an increase in the proportion of Pink stage fruit and a subsequent decrease in the proportion of Green fruit at 3 days after ethephon treatment. Immediately after ethephon application at day 1, there were 2.4-fold more DEGs compared to that at day 2, with substantially more downregulated (4.8-fold at day 1 and 2.7-fold at on day 2) than upregulated genes. Together, these results indicate that ethylene induces transcriptional changes that lead to initiation of ripening, and mainly through the downregulation of the transcriptome.

One of the mechanisms associated with ethylene-mediated progression of fruit ripening in blueberry is the downregulation of photosynthetic activity as ethephon applications immediately downregulated the expression of photosynthesis-related genes. Such downregulation of photosynthesis-related gene expression after ethephon applications were also noted in apricot (*Prunus armeniaca*) and plum (*Prunus salicina*) [51]. In contrast when 1-MCP, an ethylene perception inhibitor was applied, upregulation of genes involved in chlorophyll synthesis and photosynthesis was observed in apricot, plum and kiwifruit (*Actinidia deliciosa*) [51, 52]. 1-MCP application also de-repressed photosynthesis-related gene expression in apple fruit [33] supporting the idea that downregulation of photosynthesis is a primary component that allows for transition of the fruit into a heterotrophic organ during ripening. Together, these data support a role for ethylene in promoting fruit ripening and implicate accelerated downregulation of photosynthesis as an important component of developmental programs regulated by this phytohormone.

Anthocyanin production is regulated by a ternary complex of regulatory proteins, MYB, bHLH, and WD-40 (MBW) that coordinately increases the transcript abundance of anthocyanin biosynthesis genes [53]. In contrast, repressors of the MBW complex can negatively regulate anthocyanin production [54]. In this study *bHLH137* (PB.9591; PB.15,189) and *MYB7-like* (PB.11,494) were positively correlated, and *bHLH153* (PB.6459) and *bHLH149-like* (PB.5294) were negatively correlated with anthocyanin biosynthesis genes (Table S7). The role of ethylene in promoting anthocyanin biosynthesis is mixed. In apple, there are two regulatory modules induced by ethylene, one through the activation of MdMYB1 that promotes anthocyanin production and a second *via* MdMYB17 that negatively regulates anthocyanin biosynthesis [10, 55]. Similarly, in pear, PpERF24 and 96, regulate *PpMYB114*, to promote anthocyanin biosynthesis, whereas, PpERF105 increases the expression of *PpMYB140*, a transcriptional repressor of anthocyanin biosynthesis [11, 56]. The current study suggests that ethylene positively regulates anthocyanin production in blueberry fruit based on percent ripe (blue) fruit at day 5 after application. Since samples were collected early following ethephon treatment at day 1 and day 2, we could not identify DEGs related to anthocyanin biosynthesis after ethylene application. However, at least one regulatory transcription factor, *bHLH93-like* (PB.12,590) present in cluster 9 wherein anthocyanin biosynthesis genes were over-represented, was ethylene responsive (Table S6).

In addition to ethylene, other hormones such as ABA, auxin and JAs are known to influence ripening, often through interaction with ethylene. In the current study, ABA concentration increased during ripening reaching a peak at the Pink stage, similar to that reported in a previous study [57]. Multiple transcripts coding for *9-CIS-EPOXYCAROTENOID DIOXYGENASE*  (*NCED*) were detected in Clusters 3 and 9, suggesting that upregulation of ABA biosynthesis genes during ripening initiation between IMG and Pink stages was coincident with an increase in ABA concentration (Table S6). However, the decrease in ABA concentration between Pink and Ripe was not correlated with changes in *NCED* expression (Table S7), suggesting that other metabolic processes such as formation of an ABA conjugate, ABA-glucose ester, may have contributed to decline in ABA concentration [57]. An increase in ABA concentration during ripening has also been noted in multiple crops such as bilberry (*Vaccinium myrtillus*), strawberry, grape and tomato [17, 21, 58, 59]. Together, these data support a role for ABA in regulating the progression of ripening in blueberry. Consistently, in blueberry and other crops such as strawberry and grape, ABA applications increase the transcript abundance of genes related to anthocyanin biosynthesis and positively regulate their accumulation [60–62]. In the current study also, two *NCED* transcripts clustered with anthocyanin biosynthesis and potential regulatory genes in Cluster 9, suggesting a relationship between ABA biosynthesis and anthocyanin production (Table S6). Further, application of ABA influences cell wall modification-related genes suggesting a role in fruit softening [61, 63]. In tomato, ABA application accelerates ripening and increases the transcript abundance of ethylene biosynthesis genes, suggesting a crosstalk between ABA and ethylene in coordinating ripening [21, 22]. On the contrary when ethylene signaling was blocked using 1-MCP, ABA accumulation was delayed compared to the control suggesting that ethylene also regulates ABA accumulation [22]. Similarly, in the current study, ethephon treatment increased ABA concentration at 3 days after application suggesting that ethylene positively regulates ABA accumulation in blueberry. In support of this conclusion, in peach, ethylene induced transcription factor PpERF3, stimulated ABA biosynthesis by increasing the expression of *PpNCED2/3* [64]. Further an abscisic acid receptor *PYR1-like*, an ABA signaling gene, was upregulated as an immediate response to ethephon application suggesting possible amplification of ABA signaling. A role for the *PYR1* gene in inducing anthocyanin biosynthesis during grape ripening has been reported previously [65]. In strawberry fruit, silencing of an ABA biosynthesis gene and another receptor gene (*FaCHLH*/*ABAR*) lead to an uncolored phenotype and decrease in sugar content, respectively [59]. Together, these data support the proposition that ethylene and ABA work in concert to promote ripening during blueberry fruit development.

Free IAA concentration decreased and IAA-Asp exhibited an increasing trend during blueberry fruit ripening. Conjugated IAA-Asp, that reduces free IAA levels also increased during grape ripening [25]. Similar to our study, a pattern of decline in free IAA with an increase in IAA-Asp at ripening has been noted in other fruits such as strawberry and tomato suggesting that auxin negatively regulates fruit ripening [25, 66]. Transcriptome analysis in tomato revealed that application of auxin delays ripening by delaying ethylene production and multiple ripening-related genes [30]. However, in tomato, silencing of auxin signaling genes, *SlARF2A* and *2B* exhibited down-regulation of ripening-related genes such as ripening-related transcription factors, ethylene biosynthesis genes, cell wall metabolism gene *PG2A*, and carotenoid biosynthesis genes, indicating these genes positively regulate ripening [37]. Other studies in apple and peach also suggest that auxin-responsive transcription factors can activate ethylene production and accelerate ripening [35, 36]. In apple, MdARF5 interacts with the promoters of ethylene biosynthesis genes to promote their expression and induce production of ethylene [34]. In an apple cultivar ‘Granny Smith’, a low basal level of ethylene during ripening has been suggested to be associated with low auxin and higher IAA-Asp levels [35]. The authors suggest that a low auxin concentration in ‘Granny Smith’ may not be sufficient to stimulate ethylene production. These studies suggest that auxin may promote ripening via stimulating ethylene biosynthesis. Whether such a connection between ethylene-stimulation by auxin occurs in blueberry fruit needs further characterization.

In apple, although an auxin-responsive transcription factor positively regulates ripening, ethylene production in-turn results in a decline in auxin levels, possibly due to ethylene induced activation of the auxin conjugating enzyme, *GRETCHEN HAGEN 3 (GH3)* [35], suggesting a feedback loop between these two hormones. In the current study, ethephon application decreased the expression of auxin biosynthesis genes, *YUCCA*, and also, increased the transcript abundance of the auxin conjugating gene, *INDOLE-3-ACETIC ACID-AMIDO SYNTHETASE* (*GH3.1*). Similarly, other studies in tomato, pepper and grape support the induction of *GH3* by ethylene during fruit ripening [25, 67, 68]. Overexpression of a pepper *CcGH3* increased the rate of ripening upon ethylene treatment in tomato, suggesting this gene is ethylene-inducible [67]. In grape, the transcript abundance of *GH3* increased during ripening and was slightly upregulated in response to ethylene treatments [25]. In this study, ethylene induced transcript changes in auxin metabolism-related genes, did not translate directly to changes in IAA and IAA-Asp concentrations after ethephon application within the time period analyzed. This may be attributed in part to the high variability in IAA-Asp concentration among replicates. Hence, the potential antagonistic relationship between IAA and ethylene warrants further investigation.

Overall, there is limited information on the role of JAs in fruit ripening. While multiple studies have indicated a positive role for JAs in fruit ripening, other studies have suggested that they may delay ripening [69–72]. MeJA application promoted transcript abundance of anthocyanin biosynthesis genes and anthocyanin production in strawberry [73]. In apple, MeJA-induced TFs, MdMYB9 and MdMYB11 promoted anthocyanin accumulation [74]. However, no effect on ripening rate and fruit quality were observed after MeJA application in blueberry [75]. In the current study, JAs decreased during fruit ripening similar to that noted for IAA, suggesting a negative role for them in the regulation of fruit ripening. Further, JA concentration during ripening was negatively correlated with transcript abundance of *flavonoid 3’,5’-hydroxylase*, a gene related to anthocyanin biosynthesis, and with *pheophorbide a oxygenase*, a gene involved in chlorophyll catabolism (Table S7) suggesting that JAs may negatively regulate anthocyanin biosynthesis and chlorophyll degradation during blueberry fruit ripening. Additionally, ethephon application decreased the transcript abundance of jasmonic acid biosynthesis genes in conjunction with changes in JA levels, suggesting that ethylene downregulates JA production, potentially as part of a program associated with acceleration of ripening. Similarly, ethephon-treated apple fruit displayed downregulated activity of allene oxide synthase, a JA biosynthesis enzyme, and JA content [76].

## Conclusions

This study demonstrates that downregulation of photosynthesis is associated with ripening initiation in blueberry. Ethylene plays an important role in accelerating the onset of ripening mainly by downregulating the transcript abundance of multiple genes, especially those associated with photosynthesis. Ethylene increased ABA concentration and upregulated the transcript abundance of an ABA receptor *PYR1-like* suggesting possible amplification of ABA signaling and synergistic interaction between these phytonhormones. Free IAA concentration decreased and IAA-Asp showed an increasing trend during blueberry fruit ripening but were not significantly affected by ethylene, although, decreased transcript abundance of *YUCCA*, and increased transcript abundance of *INDOLE-3-ACETIC ACID-AMIDO SYNTHETASE*, *GH3.1* were noted. Similar to auxin, JA concentrations decreased during ripening. Ethylene decreased JA levels and the expression of JA biosynthesis genes. These results suggest an antagonistic interactions between ethylene and JAs (and possibly auxin) in regulating the progression of ripening. Together, these data present insights into mechanisms by which ethylene promotes fruit ripening in blueberry, and also offer additional avenues for further exploration, particularly in the realm of phytohormone interactions.

## Methods

### Ethephon treatment and phenotypic data collection

Rabbiteye blueberry ‘Powderblue’ plants grown at the Durham Horticulture Farm in Watkinsville, GA were used for ethephon treatments on June 23rd, 2017. Ethephon (250 mg·L^− 1^) and control treatments were applied following the method described in [75]. Each treatment had four biological replicates. Phenotypic data collected included ethylene evolution from fruit and the rate of ripening. Ethylene evolution from fruit was measured using a closed system. Approximately 25 g of fruit were harvested from each blueberry plant (biological replicates) two days after treatment and incubated in an air-tight 125 mL glass jar with a lid fitted with a rubber septum, for 4 h at room temperature. Headspace samples (1 mL) were analyzed by GC-17 A gas chromatography (GC-17 A, Shimadzu, Japan) equipped with a 2 m micropacked column (Hayesep N, Restek, PA, United States) and a flame ionization detector. The temperature of the injection port and the detector of the GC were set at 200 ^o^C. The temperature program was 60 ^o^C for 4 min; increased by 20 ^o^C.min^− 1^ to 150 ^o^C; and held at 150 ^o^C for 1 min. The peak area from the resulting chromatograph and a standard curve generated using various concentrations of ethylene were used to determine ethylene evolution from the fruit sample and expressed as nL·g^− 1^·h^− 1^. The rate of ripening was determined by visual color assessment, following the method described in [75]. In short, 3 branches were tagged on each blueberry plant, included around 100 fruits in total. The color of Green, Pink, and Ripe (blue) fruits on the 3 branches were counted when fruits were still attached to the plant at 0, 1, 2, 3, 5, 7, 10, 14 days after the treatment. Green fruit were classified as mature and appearance of color (~ 25% pink coloration) indicating ripening initiation. Pink fruit displayed predominantly pink with some blue (< 10%) and Ripe as fully blue fruit.

### RNA-sequencing

Two sets of fruit samples were collected for RNA-sequencing: (1) fruit at four developmental/ripening stages: Immature green (IMG), Green, Pink and Ripe (2) control and ethephon treated fruit at 1 and 2 days after treatment. IMG fruit was based on size ranging from > 7-<13 mm in diameter. Green, Pink, and Ripe fruit had two biological replicates, while all remaining samples had three biological replicates. Fruit from control and ethephon treatments were comprised of a random sample with fruit at varying developmental time-points. Each sample was frozen in liquid N_2_ and then stored at -80 °C until further processing. Six to eight fruit for each sample were ground into fine powder and total RNA was isolated from the tissue using the cetyltrimethyl ammonium bromide (CTAB)-based method described in [77]. High quality RNA (RIN > 8.0) from each sample was used for RNA-Seq library construction with KAPA Stranded mRNA-Seq Kit (KAPA Biosystems, USA) for the Illumina platform, following the instructions provided in the manufacturer’s manual. Libraries were sequenced at the Georgia Genomics and Bioinformatics Core at UGA using an Illumina NexSeq500 platform with 75 bp paired-end sequencing.

### RNA-seq data analyses

The reads generated by RNA-sequencing were trimmed and aligned to the full-length fruit transcriptome, with further downstream analyses to determine differential gene expression. First, adapters and low-quality reads were trimmed from the raw reads by Trimmomatic version 0.36 [78] with the parameters: LEADING:3 TRAILING:3 SLIDINGWINDOW:4:15 MINLEN:36. Afterwards, the clean reads were aligned to a full-length transcriptome of ‘Powderblue’ (generated previously in our laboratory using PacBio Sequencing [41]) by STAR version 2.7.1 [79], and the aligned reads were counted according to the genomic feature by FeatureCounts under Subread version 1.6.2 [80]. The raw counts were used for identifying differentially expressed genes (DEGs) and normalized expression level, transcripts per million (TPM), by EdgeR [81]. The cutoff of DEGs between ethephon and control treatment was 2-fold change with 0.05 false discovery rate (FDR); the cutoff of DEGs during ripening was 2-fold change with 0.01 FDR. To investigate the temporal expression patterns during ripening, all DEGs were clustered based on the standardized expression level. All DEGs during ripening were clustered based on the expression patterns by a soft clustering of time series gene expression data, Mfuzz [82]. Furthermore, GO enrichment analysis was conducted by OmicShare tools (http://www.omicshare.com/tools).

### Hormone metabolism and signaling gene identification

Genes related to ethylene, ABA, Auxin, and JA metabolism and signaling were identified from literature [83–90]. Key words for hormone metabolism and signaling genes were used to search the transcriptome. Each gene was searched for using multiple key words to ensure that all related blueberry genes were identified. These genes were also manually curated to ensure the list was comprehensive.

### Quantification of phytohormones

Hormone quantification was performed for the four developmental stages (IMG, Green, Pink and Ripe) at the Proteomics and Mass Spectrometry Facility at the Danforth Plant Sciences center (Saint Louis, MO). One hundred mg of frozen, fine ground powder from 6 to 8 fruits for each sample were used for hormone quantification. Hormones quantified during the ripening stages included ABA, IAA, IAA-Asp, JA, Ja-Ile, t-ZRiboside, c-Zeatin, t-Zeatin, OPDA, and SA. Of these only ABA, IAA, IAA-Asp, and JA displayed discernable levels during ripening. Hormone analysis of the control and ethephon treated samples at day 1, day 2 and day 3 were performed at the University of North Texas (UNT) BioAnalytical Facility. The protocol used by UNT was based on Forcat et al. [91] and quantified based on liquid chromatography with tandem mass spectrometry (LC-MS/MS). Hormones quantified included ABA, IAA, JA and their derivatives based on analysis from ripening stages. The hormones quantified included ABA, ABA-GE, IAA, IAA-Asp, JA, MeJA, Ile-JA-1, and Ile-JA-2. Ile-JA-1 and Ile-JA-2 refer to the two stereoisomers present in the standard used for quantification.

### Statistical analysis

The statistical analysis of phenotypic data and phytohormones was conducted by JMP software. To be more specific, the significant difference (α = 0.05) between the control and ethephon treated samples at each day after treatment in all phenotypic data and phytohormones was analyzed by the Student’s t-test. The significant difference (α = 0.05) of phytohormones among developmental stages were analyzed by one-way analysis of variance followed by Tukey’s HSD.

### Electronic supplementary material

Below is the link to the electronic supplementary material.

Supplementary Material 1

Supplementary Material 2

Supplementary Material 3

Supplementary Material 4

Supplementary Material 5

Supplementary Material 6

Supplementary Material 7

Supplementary Material 8

Supplementary Material 9

## Data Availability

All data supporting the findings of this study are presented in the manuscript and as supplemental data available online. The raw reads are available in the NCBI Sequence Read Archive (SRA) BioProject database (BioProject ID PRJNA976675; https://www.ncbi.nlm.nih.gov/bioproject/976675).

## Abbreviations

1-MCP: 1-Mthylcyclopropene
ABA: Abscisic Acid
ACC: 1-Aminocyclopropane-1-Carboxylic Acid
ACO: ACC Oxidase
ACS: ACC Synthase
ARF: Auxin Response Factor
bHLH: basic Helix-Loop-Helix
CTAB: Cetyltrimethyl Ammonium Bromide
DEGs: Differentially Expressed Genes
EIL1: EIN3-Like 1
ER: Endoplasmic Reticulum
ERFs: Ethylene Response Factors
GO: Gene Ontology
IMG: Immature Green
JA: Jasmonate
LC-MS/MS: Liquid Chromatography With Tandem Mass Spectrometry
MDS: Multidimensional Scaling
NCED: 9-Cis-Epoxycarotenoid Dioxygenase
PGR: Plant Growth Regulator
TA: Titratable Acidity
TPM: Transcripts Per Million
TSS: Total Soluble Solids
UNT: University of North Texas
