# Supplementary material for: Ethylene promotes fruit ripening initiation by downregulating photosynthesis, enhancing abscisic acid and suppressing jasmonic acid in blueberry (Vaccinium ashei)
Source: BMC Plant Biol. 2024 May 18;24:418. doi: 10.1186/s12870-024-05106-4 (PMC11102277; doi:10.1186/s12870-024-05106-4)
Supplement: Supplementary file 1 — Supplementary Material 1 [file 12870_2024_5106_MOESM1_ESM.docx]

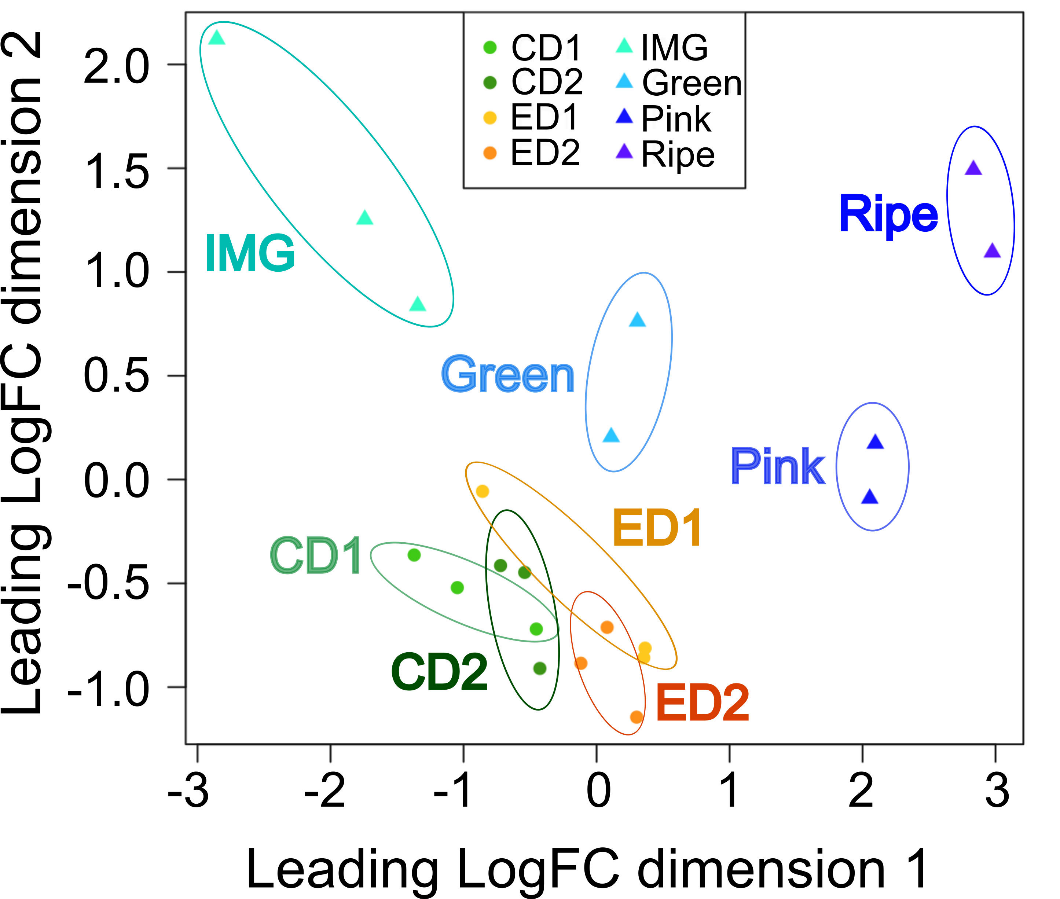


**Fig. S1** Multi-dimensional scaling (MDS) plot of rabbiteye blueberry ‘Powderblue’ fruit transcriptome using leading log2-fold-changes between each pair of samples. Samples include four ripening stages: immature green (IMG), Green, Pink, Ripe, and four samples after treatments: control day1 and day 2 (CD1, CD2), ethephon day 1 and day 2 (ED1, ED2). Each symbol represents one replicate.
