# Supplementary material for: Ethylene promotes fruit ripening initiation by downregulating photosynthesis, enhancing abscisic acid and suppressing jasmonic acid in blueberry (Vaccinium ashei)
Source: BMC Plant Biol. 2024 May 18;24:418. doi: 10.1186/s12870-024-05106-4 (PMC11102277; doi:10.1186/s12870-024-05106-4)
Supplement: Supplementary file 2 — Supplementary Material 2 [file 12870_2024_5106_MOESM2_ESM.docx]

**Fig. S2:** Top 20 enriched biological process gene ontology (GO) terms for ripening-related genes in cluster 1.

**Fig. S2.** Top 20 enriched biological process gene ontology (GO) terms for ripening-related genes in cluster 2.

**Fig. S2.** Top 20 enriched biological process gene ontology (GO) terms for ripening-related genes in cluster 3.

**Fig. S2.** Top 20 enriched biological process gene ontology (GO) terms for ripening-related genes in cluster 4.

**Fig. S2.** Top 20 enriched biological process gene ontology (GO) terms for ripening-related genes in cluster 5.

**Fig. S2.** Top 20 enriched biological process gene ontology (GO) terms for ripening-related genes in cluster 6.

**Fig. S2.** Top 20 enriched biological process gene ontology (GO) terms for ripening-related genes in cluster 7.

**Fig. S2.** Top 20 enriched biological process gene ontology (GO) terms for ripening-related genes in cluster 8.

**Fig. S2.** Top 20 enriched biological process gene ontology (GO) terms for ripening-related genes in cluster 9.
